# Drying Shrinkage Behavior and Micro-Mechanism of Concrete Based on a Thermodynamic Fractal Model

**DOI:** 10.3390/ma19142948

**Published:** 2026-07-09

**Authors:** Jianghuai Zhan, Lepeng Huang, Gang Yu, Xuanyi Xue, Ou Su, Xuran Liu, Shuai Li, Jianmin Hua

**Affiliations:** 1 School of Civil Engineering, Chongqing University, Chongqing 400045, China; 2 State Key Laboratory of Safety and Resilience of Civil Engineering in Mountain Area, Chongqing 400045, China; 3 Support and Service Center of China Academy of Engineering Physics, Mianyang 621900, China; 4 Laser Fusion Research Center, China Academy of Engineering Physics, Mianyang 621999, China; 5 China Construction Third Engineering Bureau Group Co., Ltd., Wuhan 430070, China; 6 Department of Civil Engineering, The University of Hong Kong, Pokfulam Road, Hong Kong, China

**Keywords:** low-carbon cement concrete, mechanical properties, drying shrinkage, fractal dimension, microstructure

## Abstract

This research systematically evaluated the durability performance of low-carbon cement concrete prepared with industrial solid wastes under harsh service conditions. Measurements included mechanical properties and drying shrinkage. Microstructural characterization was carried out using SEM-EDS, MIP, and TG-DTG, revealing a synergistic relationship between microstructural changes and the resulting mechanical and durability behavior of the concrete. The experimental results indicated that adding 25% fly ash (FA) lowered the compressive strength of the CF-25 and BF-25 concrete by 11.30% and 11.39%, respectively, while reducing drying shrinkage by roughly 9.2–9.5%. In comparison, incorporating 5% silica fume (SF) had contrasting effects. It significantly improved the compressive strength of the CS-5 and BS-5 concrete by 18.92% and 9.94%, respectively, but at the cost of increasing drying shrinkage by 6.30% and 18.68%, respectively. Fractal dimension analysis based on thermodynamic relationships showed that the pore structure fractal dimension (Ds) ranged from 2.88 to 2.93. Group C exhibited a higher Ds (2.93) than Group B (2.90), indicating a more intricate pore network associated with greater C-S-H gel formation. With FA addition, Ds decreased to 2.91601 for CF-25 but rose to 2.93244 for BF-25. With SF addition, Ds fell to 2.91182 for CS-5 and 2.88102 for BS-5. Micro-mechanistic analysis revealed that the limited pozzolanic activity of FA at early ages resulted in insufficient hydration products and increased porosity. In contrast, SF contributed to a dense, highly polymerized gel structure and an optimized pore size distribution through its strong pozzolanic reactivity and nano-filling action. The distinct chemical properties of high-calcium and low-calcium cementitious systems further accentuated the differential influences of these supplementary cementitious materials.

## 1. Introduction

Concrete is the most widely used man-made engineering material in the world. Its core component is ordinary Portland cement. However, producing this cement consumes high energy. It also generates large carbon emissions. This seriously hinders the achievement of the “dual carbon” goals [1,2]. Currently, the main emission reduction method relies on adding supplementary cementitious materials to traditional cement. This approach has reached a bottleneck. A fundamental change to the binder system is urgently needed. A promising alternative is new silica–alumina-based low-carbon cement. It uses industrial solid wastes as main raw materials. These wastes include fly ash, slag, and steel slag. Through mechanical activation and chemical excitation, these materials can greatly reduce or even eliminate limestone calcination. This achieves carbon reduction at the source. It also shows significant environmental advantages [3,4]. However, the drying shrinkage behavior of silica–alumina-based low-carbon cement concrete and its microstructural origin remain insufficiently understood. In particular, the effects of FA and SF on pore structure evolution, reaction product composition, and moisture-loss-induced deformation may differ substantially between ordinary Portland cement systems and low-carbon cementitious systems. This knowledge gap limits the reliable evaluation of crack resistance, dimensional stability, and engineering applicability of such low-carbon concrete.

To achieve low-carbon concrete, breakthroughs in the cementitious system are necessary. The traditional approach mainly uses industrial by-products such as FA and SF as supplementary cementitious materials. These partially replace cement [5]. FA comes from coal-fired power plants. It has pozzolanic activity. It reacts with calcium hydroxide generated by cement hydration. This reaction forms additional C-S-H gel. This helps improve the long-term strength of concrete. It also reduces hydration heat and enhances durability [6]. However, FA has low early reactivity. This often causes slow strength development at early ages. SF is a fine powder recovered from ferrosilicon and metallic silicon production. It has high silica content and a large specific surface area. Therefore, it possesses outstanding pozzolanic effect and micro-aggregate filling function. It can effectively optimize the pore structure of concrete [7]. It also significantly improves strength and durability [8,9]. Lv et al. [10] pointed out that SF is far more effective than FA in enhancing material abrasion resistance. In the GS5 sample containing 5% SF, its abrasion strength and impact energy were 18.5% and 37.9% higher, respectively, than those of the GF25 sample containing 25% FA. Wang et al. [11] found that the effect of SF on the compressive strength of geopolymer-based ultra-high performance concrete (UHPC) is complex. It is closely related to the content of calcium aluminate cement. When the calcium aluminate cement content is 20%, increasing SF dosage benefits compressive strength growth. However, when the content is 10%, too high an SF dosage (>10%) has a negative effect on strength. Chen et al. [12] showed that adding FA and SF can refine the pore structure. It reduces permeability and delays performance degradation. As a result, blended concrete exhibits slower degradation, a slower rate of porosity increase, and a longer service life compared to ordinary Portland cement concrete. Mudasir Nazeer et al. [5] confirmed that replacing 10% of ordinary Portland cement with a mixture of FA and SF significantly improves concrete strength and durability. At 28, 56, and 120 days, compressive strength increased by 41%, 85%, and 94%, respectively. Nejib Ghazouani et al. [13] found that when the SF replacement rate reaches 30%, the prepared ultra-high performance green cementitious composite achieves a 28-day compressive strength of 134.5 MPa. This SF mix also shows the smallest capillary water absorption coefficient (0.16758 mm/min^½^). Drying shrinkage decreases as SF content increases. The minimum value reaches 4000 μm/m. Thermal analysis results also show that the SF mix has lower calcium hydroxide content. Secondary hydration reactions are also stronger. Qiu et al. [14] pointed out that FA and SF have fine particle sizes and high reactivity. They help fill pores during hydration and generate more C-S-H gel. This not only improves the pore structure of the mortar. It also strengthens the interfacial transition zone around coal gangue aggregates. Although previous studies have confirmed the beneficial effects of FA and SF on strength development, pore refinement, and durability improvement, most of them focused on ordinary Portland cement systems or evaluated macroscopic properties without establishing a quantitative link between pore structure characteristics and drying shrinkage. Moreover, the roles of FA and SF may vary with the chemical nature of the binder system. In OPC-based concrete, their effects are mainly associated with clinker dilution, pozzolanic reaction, and C-S-H formation, whereas in low-carbon cementitious systems, the evolution of aluminosilicate gels, pore wall morphology, and moisture transport may become more dominant. Therefore, a simple extension of conclusions obtained from OPC systems may not be sufficient to explain the shrinkage behavior of low-carbon cement concrete.

The durability and safety of concrete structures are key factors determining their service life. Some scholars investigated the durability of structural materials after disasters [15,16,17,18]. This is especially true in harsh marine environments. Drying shrinkage is a main cause of early-age cracking in concrete. Once cracks form, they create pathways for aggressive agents like chloride ions and carbon dioxide. This significantly accelerates structural deterioration [19]. The low-carbon cement system may differ from traditional cement in terms of hydration product composition, pore structure characteristics, and moisture transport mechanisms. Therefore, an in-depth investigation of its drying shrinkage behavior is essential. This helps evaluate the crack resistance and long-term dimensional stability of concrete produced with this system.

Based on the above research gap, the scientific problem addressed in this study is how FA and SF regulate the drying shrinkage behavior of low-carbon cement concrete through changes in reaction products and pore structure characteristics, and whether this relationship can be quantitatively described using a fractal model. To answer this question, ordinary Portland cement and silica–alumina-based low-carbon cement were selected as two representative binder systems, and FA and SF were incorporated to compare their effects on mechanical properties, drying shrinkage, pore structure, and microstructural evolution. MIP was used to obtain pore structure parameters and to calculate the thermodynamic fractal dimension, while SEM-EDS and TG-DTG were employed to analyze reaction products and microstructural changes. The main original contributions of this study are as follows: (1) a thermodynamic fractal model was introduced to quantitatively characterize the pore structure of low-carbon cement concrete; (2) the relationship between fractal dimension, pore structure evolution, and drying shrinkage was established; and (3) the different mechanisms of FA and SF in high-calcium OPC and low-calcium low-carbon cementitious systems were clarified. These contributions provide a quantitative basis for understanding the microstructure–shrinkage relationship and for optimizing low-carbon concrete mixtures with improved dimensional stability.

## 2. Materials and Experimental Methods

### 2.1. Materials

**Figure 1:**
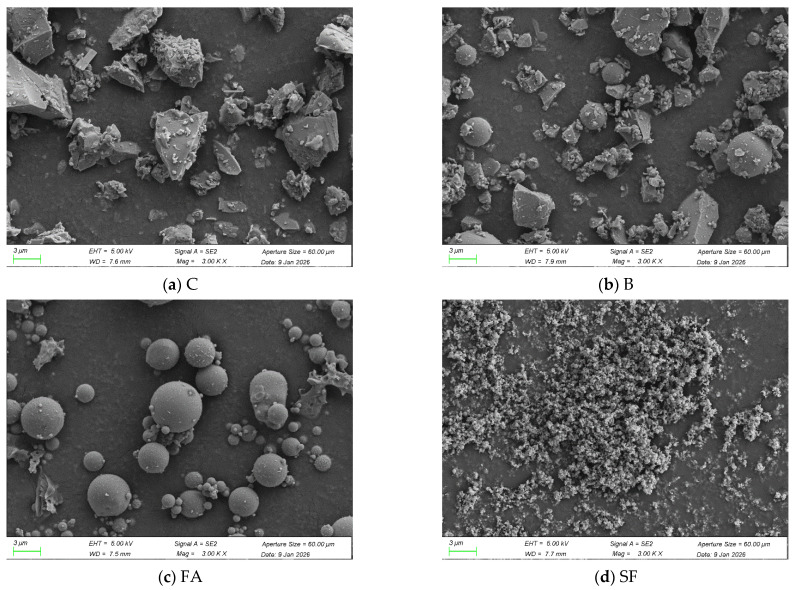
SEM images of raw materials.

**Figure 2:**
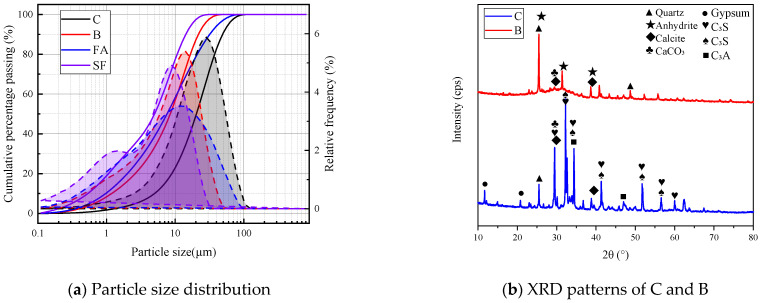
Particle size distribution and XRD of raw materials.

Two types of cementitious materials were used in this study. One was Grade P·O 42.5 ordinary Portland cement (C) from Sichuan E’sheng Cement Group Co., Ltd. (Leshan, Sichuan, China). The other was low-carbon cement (B) supplied by Shanghai Baioheng New Materials Co., Ltd. (Shanghai, China). Aggregates came from Sichuan Xinghualutong Renewable Resources Co., Ltd. (Chengdu, Sichuan, China). Manufactured sand was used as fine aggregate, and recycled aggregate served as coarse aggregate. The recycled coarse aggregate was tested for several performance indicators. Its crushing index was 11.76%. Its water absorption rate was 3.9%. Its apparent density was 2356 kg/m^3^, and its bulk density was 1578 kg/m^3^. The fly ash (FA) used in the experiments met Grade I requirements. The silica fume (SF) was a commercially available standard product. The SEM-EDS images of the cement, FA, and SF are presented in Figure 1, and their particle size distribution curves are displayed in Figure 2a. As shown in the XRD pattern in Figure 2b, the diffraction peaks of ordinary Portland cement C are mainly attributed to clinker minerals such as C_3_S, C_2_S, C_3_A, and C_4_AF, with characteristic peaks concentrated at approximately 29–34°, 41°, 47–52°, and 57–63°. Minor gypsum and calcite peaks can also be observed. In contrast, the diffraction peaks of Baioheng low-carbon cement B are mainly associated with anhydrite, calcite, quartz, and calcium aluminosilicate phases. In addition, a broad diffraction feature appears in the range of approximately 25–35°, indicating the presence of amorphous or poorly crystalline aluminosilicate components. These results demonstrate that Baioheng cement has a mineralogical composition clearly different from ordinary Portland cement, with abundant sulfate and aluminosilicate components that may participate in subsequent alkali-activated reactions.

### 2.2. Experimental Methods

#### 2.2.1. Specimen Preparation

The mix proportions of the low-carbon cement concrete used in this study are listed in Table 1. These mixture designs were adopted from our previous study on the carbonation evolution and microstructural changes of concrete containing fly ash and silica fume [20]. Six groups of concrete specimens were prepared with different binder compositions. The experimental variables included the cement type and the dosage of supplementary cementitious materials. Specifically, fly ash (FA) was used to replace 25% of the cement by mass, while silica fume (SF) was used to replace 5% of the cement by mass. The water-to-binder ratio was kept constant at 0.30 for all mixtures. Although these mix proportions have been reported previously [20], they are retained in Table 1 to provide the necessary material information for the present experimental program.

Specimen preparation followed the procedure reported in our previous work [20]. Coarse and fine aggregates were first dry-mixed for 3 min to ensure uniform distribution. The cementitious materials were then added, and mixing was continued for another 3 min. Water was added in three equal portions, with 2 min of mixing after each addition. The fresh concrete was subsequently cast into molds in layers and compacted using a vibrating table. The mold surfaces were covered with plastic film to minimize moisture loss. After 24 h at room temperature, the specimens were demolded and transferred to a standard curing environment until the predetermined testing ages.

#### 2.2.2. Mechanical Properties Tests

The mechanical properties of the concrete were tested in accordance with the Chinese standard GB/T 50081-2019 [21]. The cube compressive strength test was conducted using an electro-hydraulic servo universal testing machine with a maximum load capacity of 2000 kN. Specimens with dimensions of 100 mm × 100 mm × 100 mm were prepared for testing. The loading rate was set at 0.5 MPa/s for the compressive test. For the macroscopic mechanical tests, three parallel specimens were tested for each mixture, and the reported value was calculated as the average of the three specimens. The error bars in the corresponding figures represent the standard deviation.

#### 2.2.3. Drying Shrinkage Test

**Figure 3:**
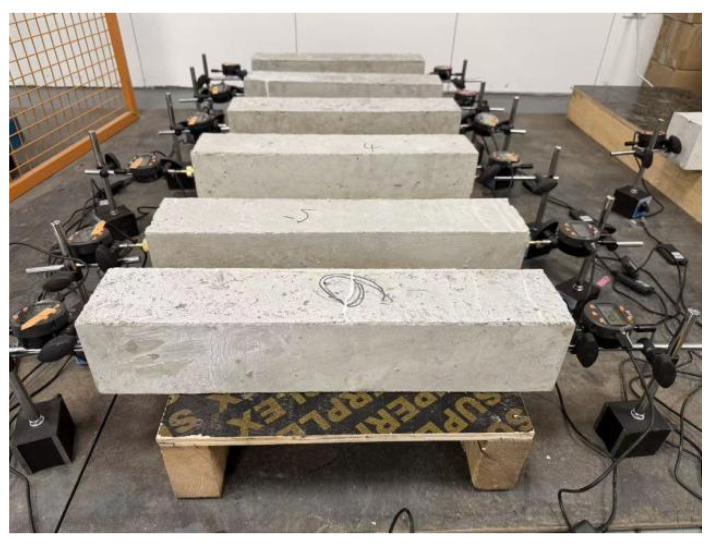
Drying shrinkage test.

The drying shrinkage test was conducted in accordance with GB/T 50082-2024 [22]. As shown in Figure 3, prism specimens with dimensions of 100 mm × 100 mm × 400 mm were used, with embedded gauge studs at both ends for length measurement. After casting, the specimens were covered with plastic film and cured under standard conditions of 20 ± 2 °C and relative humidity ≥ 95% for 24 h. The specimens were then demolded, and the initial length L_0_ was immediately measured using a length comparator with an accuracy of 0.001 mm. This value was taken as the reference length for drying shrinkage calculation. After the initial length measurement, the specimens were transferred to a drying environment of 20 ± 2 °C and relative humidity 50 ± 5%. The subsequent length measurements were conducted at 1, 2, 3, 4, 5, 6, 7, 14, 21, 28, and 56 d after demolding and the start of drying exposure. Therefore, the drying shrinkage ages reported in this study refer to the elapsed time after demolding and drying exposure. The drying shrinkage strain at each age was calculated using the formula *ε*_t_ = (*L*_0_ − *L*_t_)/*L*_b_ × 10^6^ (με), where *L*_b_ is the gauge length. For each mixture, three parallel specimens were used for the drying shrinkage test, and the reported drying shrinkage strain was the average value of the three specimens. The error bars in the drying shrinkage figure represent the standard deviation.

#### 2.2.4. Microstructural Test Method

Microstructural characterization was conducted on hardened paste specimens after 56 d of standard curing. All samples were first immersed in anhydrous ethanol to stop hydration, then crushed and dried in an oven at 60 °C for 3 d. The dried samples were ground with an agate mortar to a particle size below 80 μm for thermogravimetry-derivative thermogravimetry (TG-DTG, PerkinElmer STA 6000, Waltham, MA, USA). Additionally, fragments with particle sizes between 5 mm and 8 mm were selected for mercury intrusion porosimetry (MIP, Micromeritics AutoPore IV 9500, Norcross, GA, USA) and scanning electron microscopy (SEM, ZEISS Sigma 300, Oberkochen, Germany). For the microstructural tests, one representative sample was selected from each mixture for SEM-EDS, MIP, and TG-DTG analysis. These tests were mainly used to reveal the representative microstructural characteristics and reaction product evolution of different mixtures. For MIP analysis, the mercury contact angle was set to 130°, and the surface tension of mercury was set to 0.485 N/m. The applied pressure range of the MIP test was used to determine the corresponding pore diameter distribution according to the Washburn equation. The obtained pore size distribution and mercury intrusion data were further used to calculate the pore structure fractal dimension Ds based on the thermodynamic fractal model described in Section 3.3.

## 3. Results and Discussion

### 3.1. Mechanical Property

#### 3.1.1. Compressive Strength

**Figure 4:**
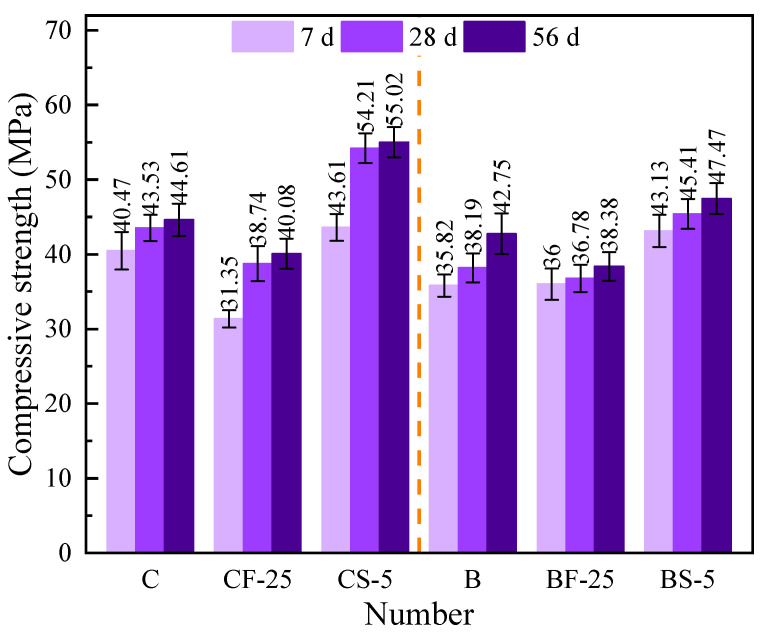
Compressive strength.

Figure 4 shows the compressive strength values and the corresponding strength growth rates for all tested specimens. For both the B-series and C-series low-carbon cement concrete mixes, adding FA had a detrimental effect on compressive strength. In contrast, incorporating SF led to higher early-age strength. Taking the 56-day curing age as an example, mix CF-25 achieved a compressive strength that was 11.3% lower than that of the plain C mix. Meanwhile, mix CS-5 showed a strength increase of 18.92% compared to the same C reference. For the B-series, mix BF-25 exhibited an 11.39% strength reduction relative to the plain B mix, whereas mix BS-5 gave a 9.94% strength improvement over the B reference. At this curing age, all low-carbon cement concrete mixtures containing 25% FA had lower compressive strengths than their corresponding control mixtures without FA. This outcome can be explained by the relatively sluggish pozzolanic reaction of FA. Within the first 56 days, FA did not fully engage in secondary hydration. As a result, its filling capability and pozzolanic activity were not yet fully mobilized to contribute to strength development. In addition, replacing part of the cement with FA lowered the clinker content in the binder system. This led to a reduced total amount of hydration products formed at early ages. By 56 d, the activation level of FA remained insufficient to offset the resulting strength loss. On the other hand, all low-carbon cement concrete mixtures containing 5% SF displayed compressive strengths higher than their respective control mixtures. This improvement is attributed to the pozzolanic action and micro-aggregate filling effect provided by SF. When comparing the two cement types, the C-series consistently outperformed the B-series in compressive strength. Moreover, the strength gain over time was more rapid in the C-series. The reason for this difference lies in the composition of each series. The C-series was dominated by highly reactive Portland cement clinker, which produced dense and strong C-S-H gel during hydration. In contrast, the B-series was based on an activated binder system made from industrial solid wastes. Its hydration products formed a weaker microstructure with lower packing density, which ultimately resulted in lower compressive strength than that of the C-series.

#### 3.1.2. Drying Shrinkage Test

**Figure 5:**
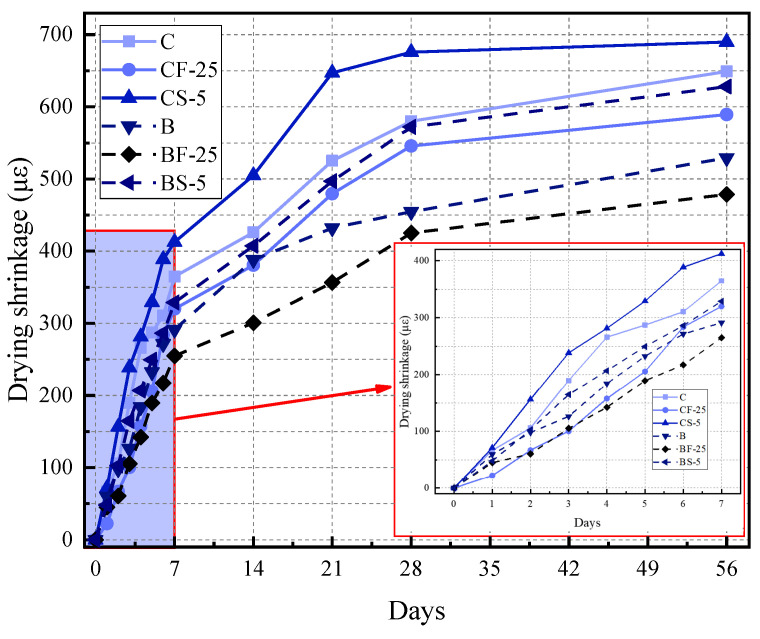
Variation in drying shrinkage.

Drying shrinkage refers to the reduction in volume of concrete during the drying and curing process. The drying shrinkage strains of low-carbon concrete under different influencing factors are shown in Figure 5. With increasing curing age, the drying shrinkage of concrete increased, with a greater increment before 7 d and a gradual stabilization thereafter. The 56 d drying shrinkage strains of the C-series concrete ranged from 589.3 to 689.9 micro-strain (με), while those of the B-series concrete ranged from 478.7 to 628.08 με. Comparing the two series, the B-series concrete exhibited lower drying shrinkage. As can be seen from Figure 5, at 56 d of curing, the drying shrinkage of CF-25 was 589.3 με, which was 9.20% lower than that of the C-group; the drying shrinkage of BF-25 was 478.7 με, representing a 9.54% reduction compared to the B-group. When cement was replaced with 25% FA, the drying shrinkage of low-carbon concrete decreased. This result was attributed to the micro-aggregate filling effect of FA, which optimized the pore structure and slowed the early-age reaction rate, thereby reducing capillary pressure and self-desiccation effects. When cement was replaced with 5% SF, the drying shrinkage of low-carbon concrete increased. At 56 d of curing, the drying shrinkage of CS-5 was 689.9 με, which was 6.30% higher than that of the C-group; the drying shrinkage of BS-5 was 628.08 με, corresponding to an 18.68% increase relative to the B-group. The increase in drying shrinkage upon 5% SF replacement may be associated with the large specific surface area and high pozzolanic activity of SF, which may increase the water demand and accelerate moisture consumption and reaction kinetics of the system. However, since fresh properties such as slump, flowability, air content, and workability retention were not measured in this study, the influence of SF on workability and effective water-to-binder ratio requires further verification. This may promote self-desiccation and capillary pore refinement, thereby contributing to the observed increase in drying shrinkage. However, this explanation should be further verified by fresh property and moisture transport tests.

### 3.2. Microstructural Analysis

#### 3.2.1. SEM-EDS Analysis

**Figure 6:**
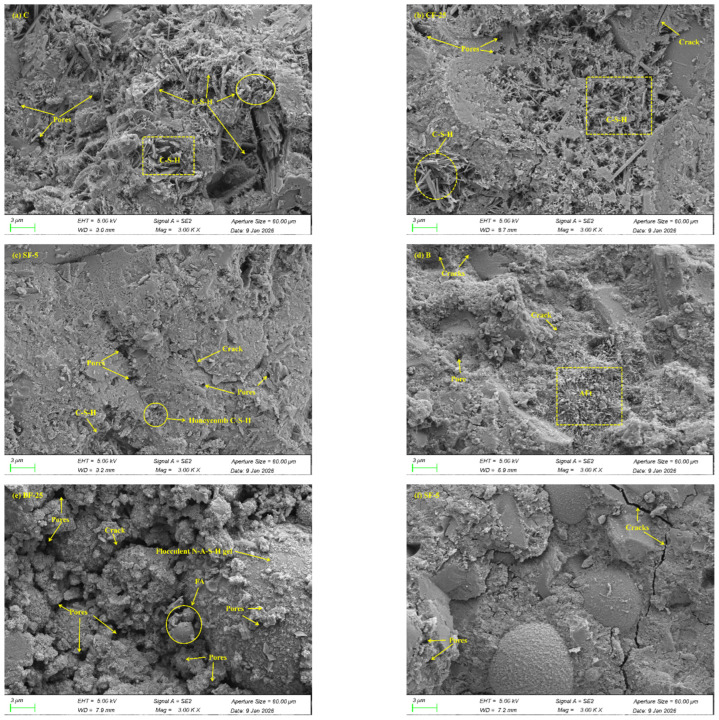
SEM analysis of different concrete samples.

SEM analysis was performed to examine the morphology of the cementitious matrix, as illustrated in Figure 6. The control mix (Group C) demonstrated a relatively dense matrix abundant in C-S-H gel, showing limited porosity and cracking. In contrast, Group CF-25 exhibited a looser matrix characterized by unreacted FA particles and a high density of pores. Group CS-5, however, formed a more compact structure. Within Group B, the reference sample showed needle-like reaction products and a gel-like matrix, which may be associated with C-S-H/C-(A)-S-H-type products and aluminosilicate gels. A marked rise in porosity was observed for Group BF-25. Conversely, Group BS-5 benefited from the filling effect of SF, which generated substantial amounts of N-A-S-H gel, leading to a comparatively dense matrix. The findings indicate that at a 25% FA replacement level, incomplete hydration reactions resulted in a loss of compressive strength. Nonetheless, FA particles contributed to filling microcracks and pores, thereby refining the pore structure. This refinement significantly lowered the drying shrinkage strain in Groups CF-25 and BF-25 relative to their respective controls. At a 5% SF replacement level, mechanical performance improved alongside a denser microstructure. However, the high specific surface area and elevated reactivity of SF made localized regions prone to forming insufficiently reacted SF agglomerates and interconnected pores. Such imperfections were responsible for the observed increase in drying shrinkage strain. When comparing the C and B systems, the N-A-S-H gel in Group B formed weaker chemical bonds with the aggregates. Moreover, this system lacked the interfacial filling and strengthening contributions typically provided by crystalline phases such as CH. As a result, the bonding within the matrix was insufficient, accompanied by higher porosity and a lower degree of hydration.

**Figure 7:**
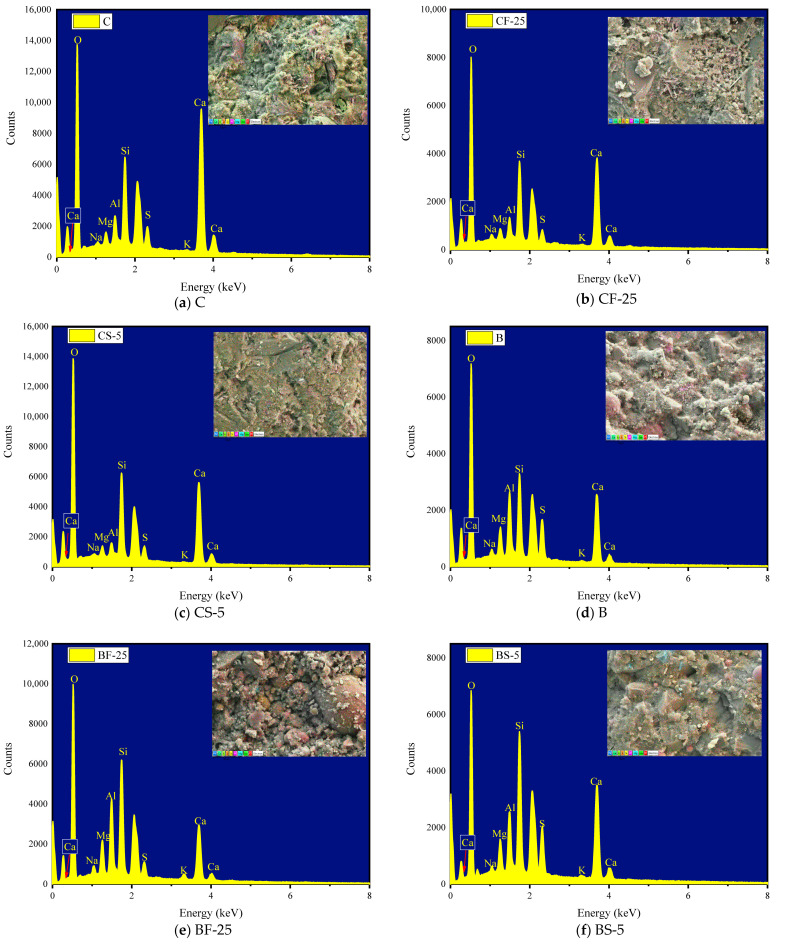
SEM-EDS analysis of different specimens.

**Figure 8:**
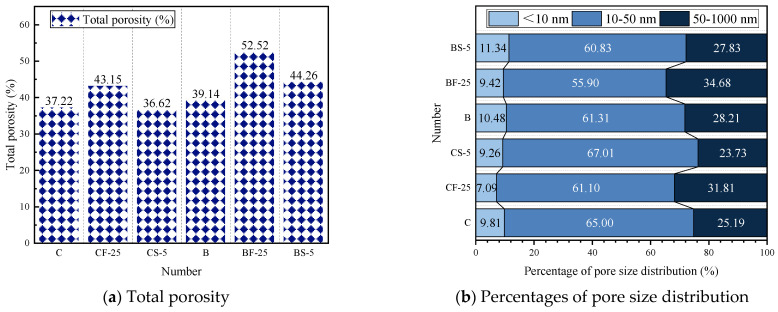
MIP analyses of different hydrated samples.

Figure 7 and Table 2 present the quantitative elemental composition and distribution of the low-carbon cement concrete paste. The elemental ratios of the reaction products were determined as follows: Si/Al ratios ranged from 1.508 to 7.774, Na/Si from 0.027 to 0.091, and Ca/Si from 1.124 to 3.867. The SEM-EDS mapping results shown in Figure 8 indicate the spatial distribution of Ca, Si, Al, and Na in the selected regions. The calculated elemental ratios suggest the possible coexistence of N-A-S-H-like aluminosilicate gels and C-S-H/C-(A)-S-H-type calcium-bearing reaction products within the matrix. However, SEM-EDS mapping alone cannot unambiguously identify the exact gel phases, and further confirmation using complementary techniques such as XRD, FTIR, or NMR is still required. Specifically, Group C recorded a Ca/Si ratio of 3.867. This value dropped to 2.653 with the addition of 25% FA (CF-25), and reached 2.911 with 5% SF (CS-5). For Group B, the Ca/Si ratio was 1.96, which decreased to 1.124 upon 25% FA incorporation (BF-25) and measured 1.602 with 5% SF (BS-5). Regarding the Si/Al ratios, Group C showed 3.568; CF-25 exhibited 4.432; CS-5 gave 7.774; Group B had 1.508; BF-25 presented 1.757; and BS-5 showed 2.628. The data clearly demonstrate that both FA and SF significantly modified the chemical composition of hydration products. Within the C system, FA reduced the Ca/Si ratio while elevating the Si/Al ratio, reflecting the dilution and consumption of calcium by the silicoaluminate components of FA. Conversely, SF dramatically increased the Si/Al ratio accompanied by a decrease in the Ca/Si ratio, highlighting the strong regulatory influence of its highly reactive SiO_2_. In the B system, which inherently possesses lower baseline Ca/Si and Si/Al values, the introduction of SCMs further lowered the Ca/Si ratio and slightly increased the Si/Al ratio; nevertheless, these values remained substantially below those observed in the C system. This observation is consistent with the reaction behavior governed by low-calcium geopolymeric gels. The observed changes in chemical composition are closely linked to the macroscopic compressive strength. Owing to its limited early-age reactivity, FA led to slow hydration product formation and inadequate microstructural densification, thereby causing a reduction in strength. In contrast, SF, through its high reactivity and micro-filling capability, may promote the formation of a denser reaction product structure and improve the compactness of the matrix, thus contributing to strength enhancement. These findings imply that the effect of SCMs on strength is governed not solely by compositional variations but also critically by their reaction kinetics and the efficiency of microstructural formation.

#### 3.2.2. MIP Analysis

Figure 8 presents the MIP analysis results for different specimens. Figure 8a indicates that the total porosities of CF-25 and CS-5 were 5.93% higher and 0.6% lower than that of C, respectively, while those of BF-25 and BS-5 were 13.88% and 5.12% higher than that of B, respectively. Comparing Groups B and C, it was observed that the total porosities in Group C were consistently lower than those in Group B. Since FA and SF possess high pozzolanic activity, they could react with calcium hydroxide during hydration to form C-S-H gel, filling pores and thereby reducing total porosity [23]. The porosities of low-carbon cement concrete mixtures containing SF were lower than those containing FA. This was attributed to the finer particle size and higher pozzolanic activity of SF. On one hand, SF particles could penetrate into pores; on the other hand, the additional C-S-H gel further filled excess pores. Consequently, this resulted in lower porosity and increased strength compared to FA mixtures.

Following reference [24], pores in hardened cement paste were classified into gel pores (d < 10 nm), medium pores (10 nm < d < 50 nm), and large pores (50 nm < d < 1000 nm). Pores smaller than 10 nm were generally considered intrinsic to C-S-H gel, while medium and large pores represented voids remaining in the water-filled space from partially hydrated cement particles, with medium pores being considered harmless. As shown in Figure 8b, FA and SF had different effects on pore size distribution. The volume of gel pores (d < 10 nm) in CF-25 and CS-5 was 2.72% and 0.55% lower than that in C, respectively, while the volume of large pores (50 nm < d < 1000 nm) was 6.62% higher and 1.46% lower, respectively. In BF-25 and BS-5, the gel pore volume was 1.06% lower and 0.86% higher than that in B, respectively, and the large pore volume was 6.47% higher and 0.38% lower than that in B, respectively. Both FA and SF refined the pore structure of the concrete through chemical reactions and physical filling effects [25]. However, at an FA replacement level of 25%, an increase in large pores was observed in the low-carbon cement concrete. This was attributed to the delayed pozzolanic reaction and the early cement dilution effect of FA, which led to a higher proportion of large pores within the standard curing period, consequently resulting in reduced strength. These MIP-derived pore structure parameters, including total porosity and the proportions of gel pores and large pores, were further used in Section 3.3 to interpret the relationship between fractal dimension and drying shrinkage.

#### 3.2.3. TG-DTG Analysis

**Figure 9:**
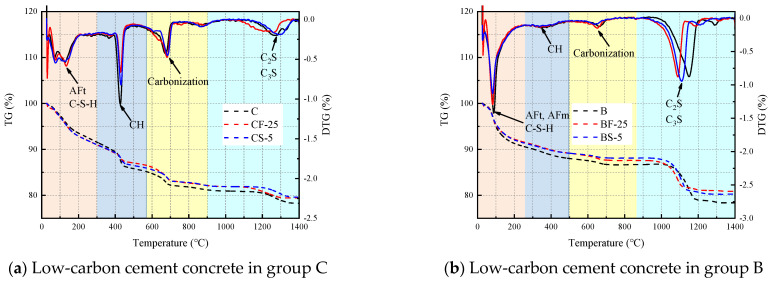
TG-DTG analysis of different hydrated samples.

Figure 9 TG-DTG curve analysis revealed the differential effects of various mineral admixtures on the two cementitious systems. In the DTG curves, the endothermic peak between 0 °C and 200 °C corresponded to the loss of free pore water and the dehydration of hydration products such as C-S-H and ettringite (ettringite decomposes around 90 °C, while C-S-H dehydration occurs between 150–400 °C) [26]. The endothermic peak in the 400–500 °C range originated from the decomposition of calcium hydroxide [27]. Peaks observed between 500–950 °C reflected the decomposition of carbonate phases, and fluctuations in the 1000–1400 °C range were potentially related to the high-temperature decomposition of calcium sulfate [28]. Comparing the C and B systems revealed distinct characteristics: the C system exhibited a significant Ca(OH)_2_ dehydration peak within 400–500 °C, reflecting its high-calcium nature, with low-temperature mass loss primarily attributed to the decomposition of C-S-H and AFt. In contrast, the B system displayed a minor Ca(OH)_2_ peak, indicating a low-calcium system where low-temperature mass loss was mainly contributed by the combined dehydration of AFt, AFm, and C-S-H. Both systems showed carbonation peaks in the high-temperature region, but differences in peak intensity reflected the varying influences of the admixtures on the carbonation process.

Within the C system, the Ca(OH)_2_ content in both CF-25 and CS-5 samples was lower than that in sample C, attributable to their pozzolanic reactions and the dilution effect on the cement content. Since the SF content in the CS-5 mixture was lower than the FA content in CF-25, the dilution effect was less pronounced, resulting in a higher Ca(OH)_2_ content in CS-5 compared to CF-25. The ultrafine particle size of SF acted as a micro-filler, occupying spaces between cement particles and reducing the porosity of the cement paste. Furthermore, the higher pozzolanic activity of SF enabled it to react with the Ca(OH)_2_ produced from cement hydration, forming more stable and higher-strength C-S-H gel [28]. In the B system, the order of Ca(OH)_2_ content from highest to lowest was: B > BF-25 > BS-5. This resulted from the distinct influences of the admixtures: sample B, with the highest clinker content, generated the most Ca(OH)_2_; sample BF-25, influenced by the dilution effect and later-stage pozzolanic reaction of FA which consumed part of the Ca(OH)_2_, exhibited the next highest content; although BS-5 had a low SF replacement rate, its high-activity pozzolanic reaction rapidly and substantially consumed Ca(OH)_2_, resulting in the lowest content.

### 3.3. Discussion on the Relationship Between the Fractal Model Based on Thermodynamic Relationships and Drying Shrinkage

The principle of the fractal model based on thermodynamic relationships is as follows [29]: When using mercury intrusion porosimetry to measure the relationship between pore volume and pore diameter of porous materials, the work done by the external environment on mercury equals the increase in surface energy of the mercury intruded into the pores. Therefore, the pressure *p* applied to mercury and the intruded volume *V* satisfy Equation (1):

(1) $$\int_{0}^{r}pdV=-\int_{0}^{s}\sigma \cos\theta dS$$

where: *σ* is the surface tension of mercury, N·m^−1^; *θ* is the contact angle between mercury and the sample, °; *S* is the pore surface area of the tested material, m^2^; *V* is the pore volume of the tested material, m^3^.

Through dimensional analysis, the fractal scaling of the pore surface area *S* of a porous material can be correlated with the pore diameter *D* and the pore volume *V*, leading to the expression of the fractal model. For the mercury intrusion stage, Equation (1) can be approximately written in a discrete form, as shown in Equation (2):

(2) $$\sum_{i=1}^{n}{\bar{p}}_{i}\Delta V_{i}=Cr_{n}^{2-D_{S}}V_{n}^{D_{S}/3}$$

where: ${\bar{p}}_{i}$ is the average mercury intrusion pressure for the *i*-th intrusion event, Pa; Δ*V_i_* is the mercury intrusion volume for the *i*-th event, m^3^; *n* is the number of pressurization steps during the mercury intrusion process; *D_n_* is the pore diameter corresponding to the *n*-th intrusion event, m; *V* is the cumulative mercury intrusion volume after *n* pressurization steps, m^3^; and *C* is a constant.

Let

(3) $$W_{n}=\sum_{i=1}^{n}{\bar{p}}_{i}\Delta V_{i},Q_{n}=V_{n}^{1/3}/D_{n}$$

then

(4) $$\mathrm{lg}(\frac{W_{n}}{D_{n}^{2}})=D_{S}\mathrm{lg}Q_{n}+\mathrm{lg}C$$

From Equations (1)–(4), it can be seen that the calculation of the pore structure fractal dimension based on mercury intrusion porosimetry can be transformed into the study of the slope of a logarithmic function relating pore volume, pore diameter, and mercury intrusion pressure. The interpretation of the correlation coefficient values in this study refers to the methods described in “MedCalc Statistical Analysis Methods and Applications” [30], as presented in Table 3.

**Figure 10:**
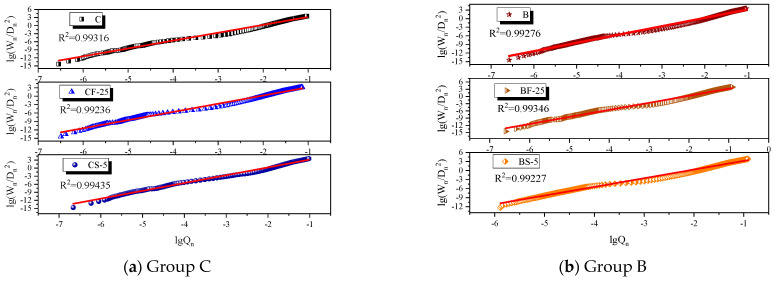
Fractal model scatter plot based on thermodynamic relations.

The pore structure scatter data obtained based on the thermodynamic model are shown in Figure 10, which reflect the pore size distribution across the entire measured pore size range, with the correlation coefficient *R*^2^ above 0.99.

As shown in Table 4, the fractal dimension of the pore structure of the low-carbon cement concrete material, derived from the fractal model based on thermodynamic relationships, ranges from 2.88 to 2.93. From the perspective of topology and fundamental concepts of fractal theory, the fractal dimension of the specimens exceeds 2.0, indicating that the pore distribution morphology is irregular and complex, which cannot be adequately described by Euclidean geometry. A higher fractal dimension corresponds to greater pore complexity, which is specifically reflected in a higher percentage of gel pores (<10 nm) and transition pores (10–100 nm) within the pore size distribution. Considering that the fractal dimension of pore structures in ordinary cement-based materials generally falls between 2.0 and 3.0, it can be concluded that the fractal model based on thermodynamic relationships exhibits good agreement. It should be noted that the differences in Ds among the mixtures were relatively small, ranging only from 2.88102 to 2.93244. Therefore, Ds was used in this study as a quantitative descriptor of pore structure complexity rather than as an independent predictor of drying shrinkage. To avoid over-interpretation, the relationship between Ds and drying shrinkage was discussed together with the MIP-derived pore structure parameters, including total porosity, gel pore proportion (d < 10 nm), and large pore proportion (50 nm < d < 1000 nm).

The comparison between the fractal dimension and drying shrinkage results indicates that drying shrinkage cannot be explained by Ds alone. For example, Group C had a higher Ds value (2.93217) than Group B (2.90240), indicating a more complex pore structure. However, the drying shrinkage of the C-series and B-series was also affected by their different binder chemistry, reaction products, and pore size distribution. After FA incorporation, the Ds value of CF-25 decreased to 2.91601, while the 56 d drying shrinkage decreased to 589.3 με. Similarly, BF-25 showed an increased Ds value of 2.93244, but its drying shrinkage decreased to 478.7 με. This indicates that the reduction in drying shrinkage caused by FA was more closely related to its micro-aggregate filling effect, slower early-age reaction, and reduced capillary pressure rather than Ds alone.

For SF-containing mixtures, CS-5 and BS-5 showed relatively low Ds values of 2.91182 and 2.88102, respectively. However, their drying shrinkage increased to 689.9 με and 628.08 με, respectively. This result indicates that a lower Ds value does not necessarily correspond to lower drying shrinkage. The increase in shrinkage after SF incorporation may be mainly related to the high specific surface area and high reactivity of SF, which promote rapid moisture consumption, capillary pore refinement, and increased capillary tension. Therefore, the influence of Ds on drying shrinkage should be interpreted together with total porosity and pore size distribution.

Combined with the MIP results in Figure 10, FA increased the proportion of large pores in both CF-25 and BF-25, whereas SF reduced the proportion of large pores in CS-5 and BS-5. These changes indicate that the pore structure evolution induced by FA and SF is complex. Ds reflects the geometric complexity of the pore network, while drying shrinkage is governed by multiple factors, including total porosity, gel pore content, large pore proportion, reaction kinetics, and moisture transport. Therefore, the fractal model provides a useful quantitative description of pore structure, but it should be used together with MIP parameters rather than as a single criterion for evaluating drying shrinkage.

## 4. Conclusions

Based on the systematic durability experiments and multi-scale analysis of industrial solid waste-based low-carbon cement concrete conducted in this study, the following conclusions were drawn:

1. Regarding compressive strength, the addition of 25% FA led to a reduction in strength. Specifically, the compressive strengths of the CF-25 and BF-25 concrete were 11.30% and 11.39% lower than those of the control groups C and B, respectively. In contrast, the inclusion of 5% SF proved effective in enhancing compressive strength. The strengths of the CS-5 and BS-5 concrete were 18.92% and 9.94% higher than those of the C and B groups, respectively, highlighting the notable early-strengthening effect of SF.
2. Regarding drying shrinkage, the incorporation of FA reduced the drying shrinkage of the concrete. The drying shrinkage values for CF-25 and BF-25 decreased by 9.20% and 9.54%, respectively. This reduction is attributed to the micro-aggregate filling effect of FA, which optimizes the pore structure. Conversely, the addition of SF increased drying shrinkage, with increases of 6.30% and 18.68% for the two respective systems. This increase is linked to the strong self-desiccation effect and the refinement of capillary pores resulting from the high reactivity of SF.
3. Analysis of the pore structure using a thermodynamic-based fractal model revealed fractal dimensions (Ds) ranging from 2.88 to 2.93. Group C exhibited a higher Ds (2.93217) compared to Group B (Ds = 2.90240), indicating a more complex and rougher pore network in the traditional cement system. This complexity likely arises from the higher content of C_3_S and C_2_S, which leads to the irregular stacking of C-S-H gel. The inherently lower fractal dimension of the Group B suggests a more uniform and smoother pore wall structure.
4. The incorporation of supplementary cementitious materials altered the relationship between fractal dimension and drying shrinkage. For Group C, adding 25% FA (CF-25) slightly reduced Ds to 2.91601, while adding 5% SF (CS-5) further lowered it to 2.91182. For Group B, FA addition (BF-25) increased Ds to 2.93244, approaching the level of Group C, whereas SF addition (BS-5) decreased Ds to 2.88102. The relationship between Ds and drying shrinkage was not governed by Ds alone. Although BF-25 showed a higher Ds value, its reduced drying shrinkage was more closely associated with the micro-aggregate filling effect of FA and the reduction in capillary pressure. In contrast, BS-5 showed a lower Ds value but a higher drying shrinkage, indicating that shrinkage was jointly affected by pore size distribution, reaction kinetics, moisture consumption, and binder characteristics.
5. Microstructural analysis using SEM-EDS, MIP, and TG-DTG indicated that the low early-age pozzolanic activity of FA results in insufficient hydration products and increased porosity, which degrades macroscopic performance. In contrast, SF, through its high pozzolanic reactivity and nano-filling effect, may promote the formation of a denser reaction product structure and refine the pore size distribution, thereby improving material properties. The distinct chemical characteristics of the two cementitious matrices (high-calcium vs. low-calcium systems) further amplify the differential effects of the FA and SF admixtures. This indicates that the influence of these materials depends not only on chemical composition changes but is also critically related to their reaction kinetics and the efficiency of microstructure formation.

## 5. Future Perspectives

In future studies, fresh properties such as slump, flowability, air content, mixture density, and workability retention should be measured. In addition, mixtures with comparable consistency should be designed by adjusting the superplasticizer dosage, so that the effects of SF reactivity, pore refinement, and workability variation on drying shrinkage can be distinguished more clearly.

## Figures and Tables

**Table 1:** Mix proportions of low-carbon concrete.

Number	C (kg/m^3^)	B (kg/m^3^)	FA (kg/m^3^)	SF (kg/m^3^)	Sand (kg/m^3^)	Recycled Coarse Aggregate (kg/m^3^)	Water (kg/m^3^)	Water-Reducing Admixture (kg/m^3^)
C	500	0	0	0	678	1106	150	2
CF-25	375	0	125	0	678	1106	150	2
CS-5	475	0	0	25	678	1106	150	2
B	0	500	0	0	678	1106	150	2
BF-25	0	375	125	0	678	1106	150	2
BS-5	0	475	0	25	678	1106	150	2

Note: C denotes ordinary portland cement, B denotes low-carbon cement; CF indicates the addition of FA, while CS indicates the addition of SF. For example, CF-25 represents 25% cement replacement by FA, and BF-25 represents 25% low-carbon cement replacement by FA.

**Table 2:** Elemental component results of representative EDS spectra from Figure 7.

Number	Elemental Component (At%)									Element Ratio		
	O	Na	Mg	Al	Si	S	K	Ca	Fe	Si/Al	Na/Si	Ca/Si
C	61.08	0.3	0.96	1.85	6.6	2.4	0.18	25.52	1.11	3.568	0.045	3.867
CF-25	64.84	0.62	1.11	1.76	7.8	2.01	0.23	20.69	0.94	4.432	0.079	2.653
CS-5	62.08	0.22	1	1.06	8.24	2.1	0.14	23.99	1.17	7.774	0.027	2.911
B	61.34	0.73	2.63	5.31	8.01	5.6	0.23	15.7	0.46	1.508	0.091	1.960
BF-25	59.5	1.03	3.3	6.72	11.81	2.46	0.99	13.28	0.91	1.757	0.087	1.124
BS-5	54.79	0.52	2.54	4.43	11.64	5.71	0.37	18.65	1.35	2.628	0.045	1.602

**Table 3:** Significance of correlation coefficient.

Absolute Value of Correlation Coefficient	Correlation Strength	Correlation
0.9~1.0	Very strong correlation	Correlation
0.7~0.9	Strong correlation	
0.5~0.7	Weak correlation	
0.5<	Very weak correlation	Uncorrelation

**Table 4:** Fractal dimension of low-carbon cement concrete.

Number	Thermodynamic Model	
	*R* ^2^	Fractal Dimension *D_s_*
C	0.9991	2.93217
CF-25	0.99839	2.91601
CS-5	0.99838	2.91182
B	0.9987	2.9024
BF-25	0.99834	2.93244
BS-5	0.99831	2.88102

## Data Availability

The original contributions presented in this study are included in the article. Further inquiries can be directed to the corresponding author.
